# Perioperative Multi-Kingdom Gut Microbiota Alters in Coronary Artery Bypass Grafting

**DOI:** 10.3390/biomedicines13020475

**Published:** 2025-02-14

**Authors:** Zhou Fu, Yanxiong Jia, Jing Zhao, Yulin Guo, Boqia Xie, Kun An, Wen Yuan, Yihang Chen, Jiuchang Zhong, Zhaohui Tong, Xiaoyan Liu, Pixiong Su

**Affiliations:** 1 Department of Cardiovascular Surgery, Beijing Chao-Yang Hospital, Capital Medical University, Beijing 100020, China; 2 Heart Center and Beijing Key Laboratory of Hypertension, Beijing Chao-Yang Hospital, Capital Medical University, Beijing 100020, China; 3 Medical Research Center, Beijing Institute of Respiratory Medicine and Beijing Chao-Yang Hospital, Capital Medical University, Beijing 100020, China; 4 Department of Cardiology, Beijing Chao-Yang Hospital, Capital Medical University, Beijing 100020, China; 5 Department of Respiratory and Critical Care Medicine, Beijing Institute of Respiratory Medicine and Beijing Chao-Yang Hospital, Capital Medical University, Beijing 100020, China

**Keywords:** coronary artery disease, coronary artery bypass grafting, gut microbiota

## Abstract

**Background**: Coronary artery bypass grafting (CABG) is one of the main treatments for coronary heart disease (CHD). Gut microbiota, including bacteria, fungi, archaea, and virus, has been reported to be associated with CHD. However, the changes in the multi-kingdom gut microbiota after CABG are not yet clear. This study aimed to explore the changes in multi-kingdom gut microbiota during the early postoperative period of CABG. **Methods**: We collected fecal samples from 40 patients before and 1 week after CABG surgery. Metagenomic sequencing was used to detect the microbial spectrum and gene functions in the patients’ fecal samples. **Results**: Post-CABG patients exhibited significant changes in the composition of multi-kingdom gut microbiota and gene functions. Among bacteria, beneficial species such as *Bifidobacterium*, *Bacteroides*, and *Blautia* were significantly reduced after CABG, while the harmful species *Enterococcus* was significantly increased. In fungi, *Schizosaccharomyces pombe* was significantly decreased in the postoperative group, while *Saccharomyces cerevisiae* and *Aspergillus chevalieri* were significantly increased postoperatively. Spearman correlation analysis indicated that *Schizosaccharomyces pombe* had positive interactions with beneficial bacteria such as *Lachnospiraceae*, *Ruminococcus*, and *Blautia*. Among archaea, the preoperatively enriched *Methanomethylovorans-SGB40959* was significantly reduced postoperatively, and Spearman correlation analysis showed a significant positive interaction with probiotics *Ruminococcus* and *Dorea*. In viruses, the phage *Enterococcus virus EFP01*, which infects *Enterococcus*, was significantly increased postoperatively and showed a significant positive interaction with *Enterococcus*. Additionally, postoperative dysregulation of gene functions such as the Phosphoenolpyruvate-dependent Sugar Phosphotransferase System (PTS), Transposition, DNA-mediated, and Transposase Activity was observed, and Spearman correlation analysis indicated significant correlations between the dysregulated gene functions and the microbial communities. **Conclusions**: This study comprehensively revealed the changes in multi-kingdom species post-CABG. The reduction of beneficial microorganisms and the increase of harmful microorganisms after surgery are of significant clinical importance for understanding the overall health status of post-CABG patients and for optimizing postoperative treatment plans. Future research needs to further explore how to improve the prognosis of post-CABG patients by modulating the gut microbiota.

## 1. Introduction

Coronary artery disease is one of the most common causes of death worldwide. As identified by the World Health Organization [1], statistics show that over 17 million people globally die from coronary artery disease each year [2]. Coronary artery bypass grafting (CABG) is the standard treatment for coronary artery disease, particularly for left main or triple-vessel coronary artery disease [3].

The gut microbiota, which includes bacteria, viruses, fungi, and archaea, is often referred to as a ’hidden organ’. In a healthy adult, the gut microbiota consists of more than 1000 species and has a population of 10 to 100 trillion, which is about 10 times the total number of the human’s own cells. The genetic information contained within the gut microbiota exceeds that of the entire human genome by more than 150 times. The microbiota plays a crucial role in fermenting food, defending against pathogens, maintaining gut mucosal function, and regulating the immune system [4,5].

Different types of surgery lead to distinct patterns of change in the intestinal microbiome. Coronary artery bypass grafting (CABG) is unique in this regard. During CABG, a significant amount of anticoagulants is required to prevent blood coagulation, maintaining a relatively active blood flow. Moreover, the surgical procedure induces substantial hemodynamic changes, particularly during vascular anastomosis and heart manipulation, which significantly impact gastrointestinal blood flow. These rapid alterations affect the intestinal mucosal barrier and result in a cascade of nutritional and metabolic changes in the intestinal flora.

The specific alterations in multi-kingdom gut microbiota following CABG surgery remain unclear. In this study, fecal samples from 40 patients undergoing CABG were collected preoperatively and postoperatively. Metagenomic sequencing was utilized to assess the taxonomic and functional shifts in the gut microbiota. By highlighting the key microbial changes, we aim to provide comprehensive insights of how the intestinal microbiota changes after CABG surgery.

## 2. Materials and Methods

### 2.1. Study Population

This study included 40 patients from the Department of Cardiovascular Surgery at our hospital who were scheduled for coronary artery bypass grafting (CABG). We enrolled the patients with coronary artery disease who were at least 18 years old and scheduled for CABG. Antibiotic use for more than three days within the past 1 month, a history of arrhythmias (such as atrial fibrillation and ventricular tachycardia), malignancies, genetic disorders, autoimmune diseases, a history of gastrointestinal surgery, and chronic intestinal diseases or Crohn’s disease were among the exclusion criteria.

Upon admission, demographic information, including the patient’s name, gender, age, medical history, and personal habits such as smoking and alcohol use, was collected through interviews. Physical examination data, such as height, weight, and blood pressure, were documented. Additionally, laboratory test results—the first day of hospitalization and the seventh day after surgery—were retrieved from the hospital’s electronic information system. These tests included blood routine analysis, biochemistry, troponin I (TNI), and brain natriuretic peptide (BNP). The systemic immune-inflammation index (SII) was calculated as follows: SII = Platelet Count (P) × Neutrophil-to-Lymphocyte Ratio (N/L). This study was approved by the hospital’s ethics committee (2024-ke-754), and informed consent was obtained from all participants.

### 2.2. Sample Collection

Fecal samples were collected on the second day after admission and 3–7 days postoperatively to analyze changes in gut microbiota. Blood samples were collected on the second day of hospitalization and on the seventh day after surgery. Upon enrollment, each participant was provided with a sterile fecal sampling kit and given detailed instructions on the proper procedure for collecting fecal samples. Participants were instructed to deposit the stool into a sterile bedpan and, after donning gloves, use the sterile fecal sampling tool to collect a sample from the middle portion of the stool (as the outer layer may contain sloughed intestinal mucosal cells and is more prone to contamination). The collected sample was then placed into a 1.5 mL sterile Eppendorf tube and properly labeled. The fecal samples were immediately stored at 4 °C and transferred to −80 °C within one hour for storage until analysis.

### 2.3. Metagenomic Sequencing Data Analysis and Functional Annotation of Species

#### 2.3.1. DNA Extraction and Library Construction from Fecal Samples

For samples that passed initial quality checks, DNA was extracted by Oriental Yeekang (Beijing) Pharmaceutical Technology Co., Ltd. (Beijing, China) using QIAamp DNA Stool MiniKit (Qiagen, Hilden, Germany, A29790), following the manufacturer’s instructions. The extracted DNA was then tested for quality. DNA samples that met quality standards were randomly fragmented into approximately 350 bp fragments using a Covaris ultrasonic processor (Covaris, LE220R-plus, Woburn, MA, USA). The entire library preparation process was completed through end repair, A-tailing, adapter ligation, purification, and PCR amplification.

Once the library construction was completed, a preliminary quantification was performed using Qubit 3.0, and the library was diluted to a concentration of 2 ng/µL. The insert size of the library was then assessed using an Agilent 5400 Bioanalyzer (Santa Clara, CA, USA). If the insert size met the expected range, the library’s effective concentration was accurately quantified using a Q-PCR method (with an effective library concentration >3 nM) to ensure the quality of the library.

#### 2.3.2. Sequencing and Data Analysis

After passing the library quality check, different libraries were pooled according to their effective concentrations and target data output requirements, followed by sequencing on the Novaseq X Plus (Illumina, San Diego, CA, USA). The raw data obtained from sequencing may contain a certain proportion of low-quality data. The raw data were subjected to quality control (QC) to guarantee the precision and dependability of further data analyses. This involved removing reads with a high proportion of low-quality bases (quality value ≤ 30) exceeding a specified threshold (default set at 50 bp), removing reads with an excessive proportion of N bases (default set at 10 bp), and eliminating reads with overlap between adapters exceeding a certain threshold (default set at 15 bp). These first three steps were performed using the Trimmomatic tool. Additionally, if there was host contamination in the samples, host reads were filtered out by aligning against a host database using KneadData software (version 0.12.0), resulting in clean data.

#### 2.3.3. Taxonic and Functional Annotation

Starting from the clean data, MetaPhlAn4 software (version 4.0.6 (1 March 2023)) was used to compare with the mpa_vOct22_CHOCOPhlAnSGB_202212 microbial gene database to obtain species abundance tables across different taxonomic levels, including archaea and bacteria. For fungi and virus, Kraken2 (version 2.1.3) and Bracken2 (version 2.9) software were utilized to compare with the NCBI RefSeq Fungi and Viral microbial gene database to generate fungal and virus species abundance tables across various taxonomic levels. The functional annotation of genes, metabolic pathways, and resistance genes was conducted using HUMAnN3 software (humann v3.8), which mapped the data against the MetaCyc metabolic pathways database, the UniProt gene and protein database, and the CARD (Comprehensive Antibiotic Resistance Database). The output included abundance information for genes, metabolic pathways, and resistance genes, with connections to the GO, MetaCyc, and CARD databases.

### 2.4. Statistical Analysis

Using the species abundance matrix for bacteria, fungi, archaea, and viruses, the alpha diversity of species was analyzed using the vegan package in R (v4.3.3). This analysis included indices such as Shannon, Simpson, inverse Simpson, species richness, and evenness. The alpha diversity between groups was compared using GraphPad Prism (v10.1.1). For data following a normal distribution, paired *t*-tests were used for comparison, while non-normally distributed data were compared using the Wilcoxon rank-sum test. Beta diversity changes and functional gene variations between pre- and post-surgery groups, at the phylum, genus, and species levels, were analyzed using Principal Component Analysis (PCA) and Non-Metric Multi-Dimensional Scaling (NMDS) in R, with Bray–Curtis distances used to calculate adonis R^2^ and *p*-values. The ggplot2 package in R was used to analyze and visualize the top 10 most abundant species at the phylum, genus, and species levels for bacteria, fungi, archaea, and viruses. Differences in species between groups were detected using the rank-sum test, and the impact of differential species was assessed through Linear Discriminant Analysis (LefSe) to obtain LDA score distribution histograms. Spearman correlation analysis between bacteria and fungi, archaea, and viruses, as well as between species and gene functions, was performed using the psych package in R. Correlation heatmaps were generated, with a *p*-value of less than 0.05 considered statistically significant.

## 3. Results

### 3.1. Clinical Characteristics of the Study Population

A total of 40 patients with coronary atherosclerotic heart disease scheduled for coronary artery bypass grafting (CABG) were included. Among them, 20 patients underwent off-pump coronary artery bypass grafting (OPCAB), and another 20 patients underwent hybrid coronary revascularization (HCR). All patients received prophylactic antimicrobial treatment after surgery. The antibiotics used were beta-lactams. The basic information of the patients is shown in Table 1.

Patients showed an increasing trend in systemic immune-inflammatory index (SII) and plasma white blood cells (WBC), neutrophils (NE) after surgery, while lymphocytes (LY) decreased postoperatively, indicating an enhanced systemic inflammatory response and decreased immune capacity following CABG. Liver function indicators, aspartate aminotransferase (AST), and alanine aminotransferase (ALT) levels significantly increased after surgery; renal function indicators, serum creatinine, and blood urea levels also significantly increased postoperatively, while serum uric acid levels significantly decreased. Cardiac function indicator, cardiac troponin I (cTnI), significantly increased after surgery, as shown in Table 2.

### 3.2. Changes in Gut Microbiology After Surgery

**Figure 1:**
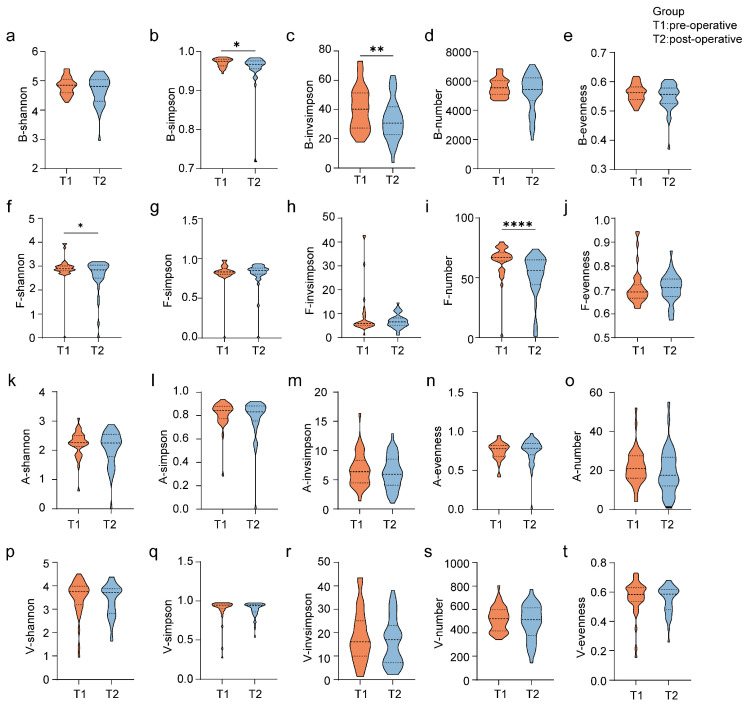
Postoperative changes in Alpha diversity. Alpha diversity of multi-kingdom microbiota before and after surgery, as represented by Shannon index, Simpson index, inverse Simpson index, species richness, and evenness. (**a**–**e**) Bacteria; (**f**–**j**) Fungi; (**k**–**o**) Archaea; (**p**–**t**) Virus. * *p* < 0.05; ** *p* < 0.01; **** *p* < 0.0001.

**Figure A1:**
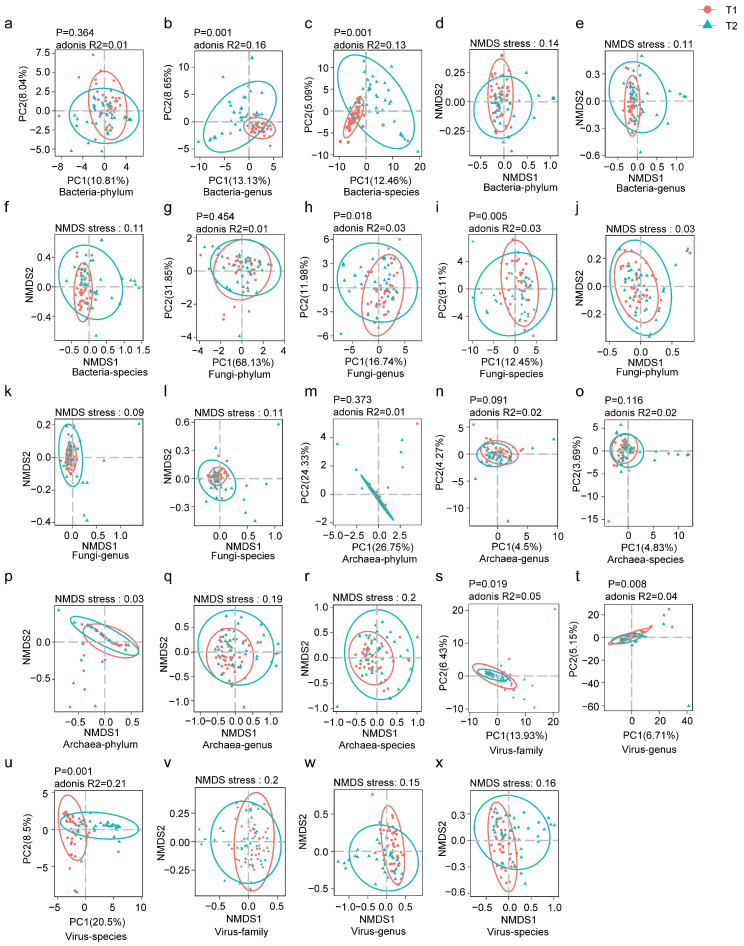
Postoperative Changes in Beta diversity. Changes in beta diversity at phylum, genus, species levels of bacteria, fungi, archaea and family, genus, species levels of virus based on principal component analysis (PCA) and non-metric multidimensional scaling (NMDS). (**a**–**f**) Bacteria; (**g**–**l**) Fungi; (**m**–**r**) Archaea; (**s**–**x**) Virus. PCA: The x–axis and y–axis represent the first and second principal components, respectively, with percentages indicating their contribution to the total variance between samples. Each point represents an individual sample, and samples from the same group are color-coded. Bray–Curtis distances were used to calculate Adonis R^2^ and *p* values. NMDS: Each point represents a sample, and the distance between points reflects the degree of dissimilarity between samples. Samples from the same group are color-coded. A stress value of less than 0.2 indicates acceptable reliability for the NMDS results. T1 represents preoperative group and T2 represents postoperative group.

To evaluate the changes in the diversity of multi-kingdom microbial communities in the gut after coronary artery bypass grafting (CABG) surgery, we initially assessed the alpha diversity of preoperative and postoperative microbial communities using the Shannon index, Simpson index, inverse Simpson index, richness, and evenness. Through paired sample *t*-tests, we observed a significant decrease in the Simpson and Shannon index of bacterial and fungi communities postoperatively (Figure 1b,c,f,i). The richness and evenness of archaea, and viruses did not significantly change after surgery (Figure 1f–t). Subsequently, we utilized PCA and NMDS to evaluate the differences in microbial species composition between the preoperative and postoperative groups. The results indicated that the composition of bacteria and fungi at the genus and species levels significantly changed after surgery (Figure A1b,c,h,i). Before surgery, the microbial communities were closely clustered, whereas, after surgery, the microbial composition only partially overlapped with that before surgery (Figure A1b,c), becoming more dispersed and significantly altered (*p* < 0.05) (Figure A1b,c). No significant changes were observed in the species composition of archaea at the phylum, genus, and species levels (Figure A1 m–r). In contrast, the species composition of viruses at the family, genus, and species levels underwent significant changes (Figure A1s–x). Finally, by calculating the relative abundance and performing LefSe analysis, we identified the predominant and significantly changing species in the postoperative groups, as detailed below.

#### 3.2.1. Bacterial Changes After Surgery

**Figure 2:**
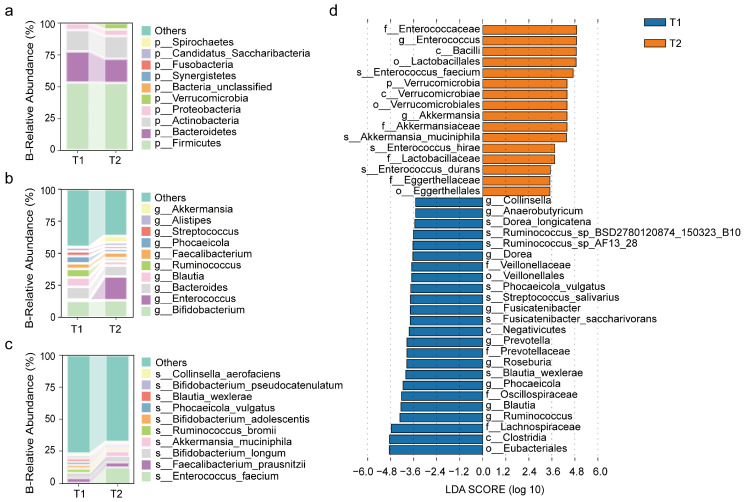
Postoperative Changes in Abundance of Bacteria. (**a**–**c**) Relative abundance of the top ten most common bacterial species at the phylum, genus, and species levels before (T1) and after surgery (T2). The vertical axis shows species abundance, and the horizontal axis shows the preoperative (T1) and postoperative (T2) groups. (**d**) Results from Linear Discriminant Analysis (LefSe), highlighting bacterial species with an LDA score greater than 3.5 at various taxonomic levels (phylum to species) between preoperative and postoperative groups.

At the phylum level, *Firmicutes*, *Bacteroidetes*, *Actinobacteria*, and *Proteobacteria* were the dominant bacterial phyla in both the preoperative and postoperative groups (Figure 2a). At the genus level, the bacteria with higher abundance in the preoperative group were *Bifidobacterium*, *Bacteroides, Blautia*, *Ruminococcus*, and *Faecalibacterium*. In the postoperative group, the relative abundance of *Enterococcus* significantly increased, becoming the most dominant genus (Figure 2b,d). At the species level, the species with higher abundance in the preoperative group were *Faecalibacterium prausnitzii*, *Bifidobacterium longum*, *Ruminococcus bromii*, and *Bifidobacterium adolescentis*, while in the postoperative group, the species with higher abundance were *Enterococcus faecium*, *Akkermansia muciniphila*, *Bifidobacterium longum*, and *Faecalibacterium prausnitzii* (Figure 2c,d).

#### 3.2.2. Changes in Fungal Communities After Surgery

**Figure 3:**
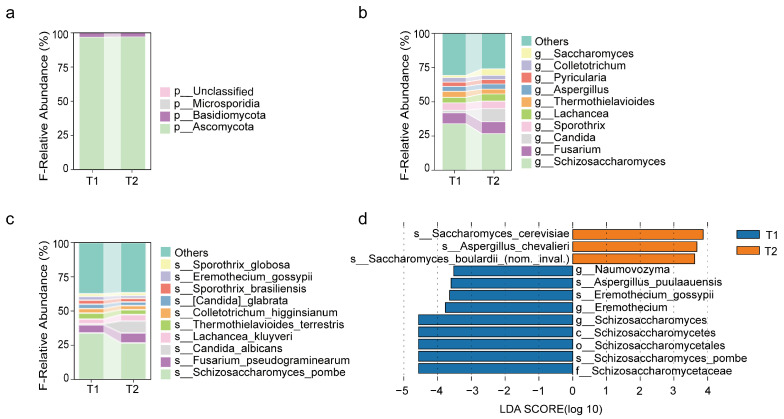
Postoperative changes in abundance of fungi. (**a**–**c**) Relative abundance of the top ten most common fungal species at the phylum, genus, and species levels before (T1) and after surgery (T2). The vertical axis shows species abundance, and the horizontal axis shows preoperative (T1) and postoperative (T2) groups. (**d**) Linear Discriminant Analysis (LefSe) highlighting fungal species with an LDA score greater than 3.5, comparing the preoperative and postoperative groups across taxonomic levels from phylum to species.

**Figure 4:**
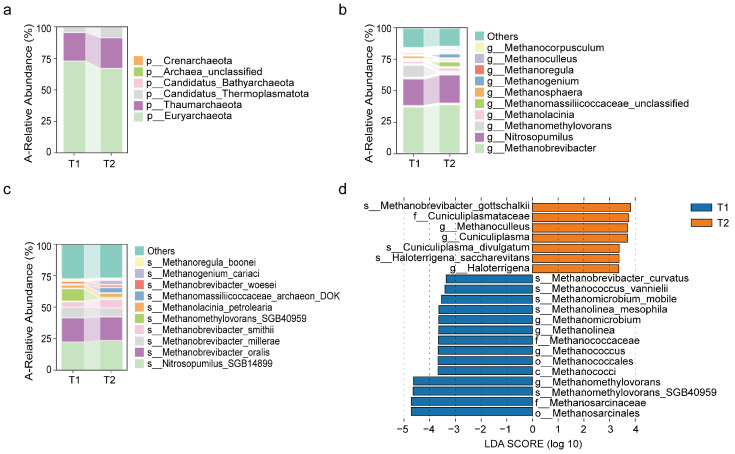
Postoperative changes in abundance of archaea. (**a**–**c**) Relative abundance of the top ten most common archaeal species at the phylum, genus, and species levels before (T1) and after surgery (T2). The vertical axis shows species abundance, and the horizontal axis shows preoperative (T1) and postoperative (T2) groups. (**d**) Linear Discriminant Analysis (LefSe) highlighting archaeal species with an LDA score greater than 3.3, comparing the preoperative and postoperative groups across taxonomic levels from phylum to species.

At the phylum level, the most abundant group in the preoperative group was *Ascomycota*, followed by *Basidiomycota* (Figure 3a). At the genus level, the genera with higher abundance in both the preoperative and postoperative groups were *Schizosaccharomyces*, *Fusarium*, *Sporothrix*, *Lachancea*, *Thermothielavioides*, *Aspergillus*, and *Pyricularia*. Notably, *Candida* and *Saccharomyces* also showed higher abundance in the postoperative group (Figure 3b), while *Schizosaccharomyces* significantly decreased in abundance after surgery (Figure 4d). At the species level, the top ten species in terms of abundance in both the preoperative and postoperative groups were *Schizosaccharomyces pombe*, *Fusarium pseudograminearum*, *Lachancea kluyveri*, *Thermothielavioides terrestris*, *Colletotrichum higginsianum*, *Candida glabrata*, *Sporothrix brasiliensis*, *Eremothecium gossypii*, and *Sporothrix globosa*. *Schizosaccharomyces pombe* significantly decreased in the postoperative group. (Figure 3c,d).

#### 3.2.3. Changes in Archaeal Communities After Surgery

At the phylum level, the archaea with higher abundance in both the preoperative and postoperative groups were *Euryarchaeota* and *Thaumarchaeota*, as well as *Candidatus Thermoplasmatota* (Figure 4a). At the genus level, the genera with higher abundance in both groups were *Methanobrevibacter*, *Nitrosopumilus*, and the *methylotrophic methanogen genus* (Figure 4b). It is noteworthy that the abundance of the *methylotrophic methanogen genus* significantly decreased in the postoperative group (Figure 4d). At the species level, the species with higher abundance in both the preoperative and postoperative groups were *Nitrosopumilus SGB14899*, *Methanobrevibacter oralis*, *Methanobrevibacter millerae*, and *Methanobrevibacter smithii* (Figure 4c). We observed that *Methanomethylovorans SGB40959* had higher abundance in the preoperative group and significantly decreased in the postoperative group (Figure 4c,d).

#### 3.2.4. Changes in Viral Communities After Surgery

**Figure 5:**
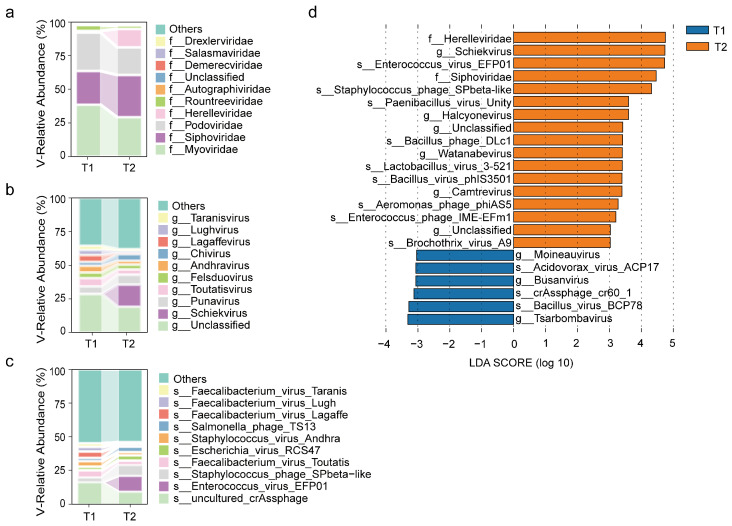
(**a**–**c**) Relative abundance of the top ten viral taxa at the family, genus, and species levels before (T1) and after surgery (T2). The vertical axis represents the relative abundance of taxa, and the horizontal axis represents the preoperative (T1) and postoperative (T2) groups. (**d**) Linear Discriminant Analysis (LefSe) highlighting differential viral taxa with LDA scores greater than 3.3, comparing preoperative and postoperative groups across taxonomic levels from phylum to species.

At the family level, viruses with higher abundance in both the preoperative and postoperative groups were *Myoviridae*, *Siphoviridae*, and *Podoviridae*, while *Herelleviridae* showed a notable increase in the postoperative group (Figure 5c,d). At the genus level, viruses with higher abundance in both groups were *Punavirus*, *Toutatisvirus*, *Felsduovirus*, and *Andhravirus*, whereas the abundance of *Schiekvirus* significantly increased after surgery according to LefSe analysis (Figure 5c,d). At the species level, viruses with higher abundance in both the preoperative and postoperative groups were *uncultured crAssphage*, *Staphylococcus phage SPbeta-like*, and *Faecalibacterium virus Toutatis*, while viruses with increased abundance after surgery and identified by LefSe analysis were *Enterococcus virus EFP01* and *Staphylococcus phage SPbeta-like* (Figure 5c,d).

#### 3.2.5. Changes in Genes, Metabolic Pathways, and Resistance Genes After Surgery

**Figure 6:**
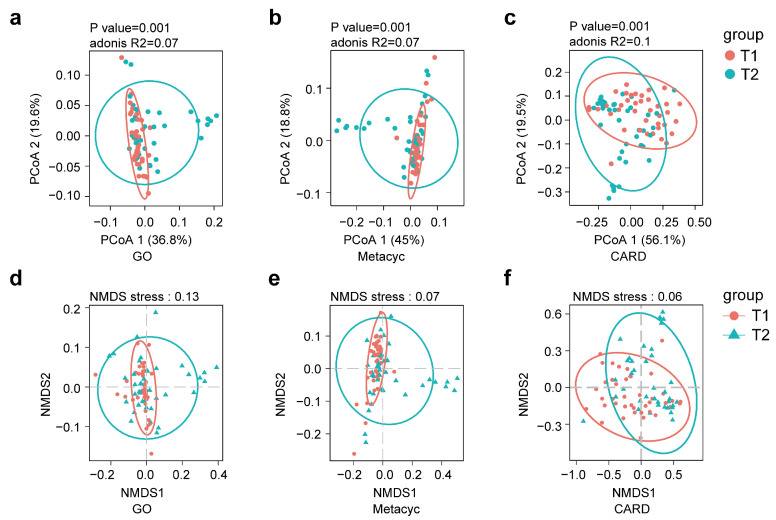
Differences in GO genes, MetaCyc pathways, and CARD genes between T1 (preoperative) and T2 (postoperative) groups. (**a**–**c**) Principal coordinates analysis (PCoA) based on Bray–Curtis distances showing differences in GO genes, MetaCyc pathways, and CARD genes between T1 and T2. PCoA1 and PCoA2 represent the two components with the greatest variance, and the percentages indicate their contribution to total variance. (**d**–**f**) Non-metric multidimensional scaling (NMDS) showing differences in GO terms, MetaCyc pathways, and CARD genes between T1 and T2.

**Figure A2:**
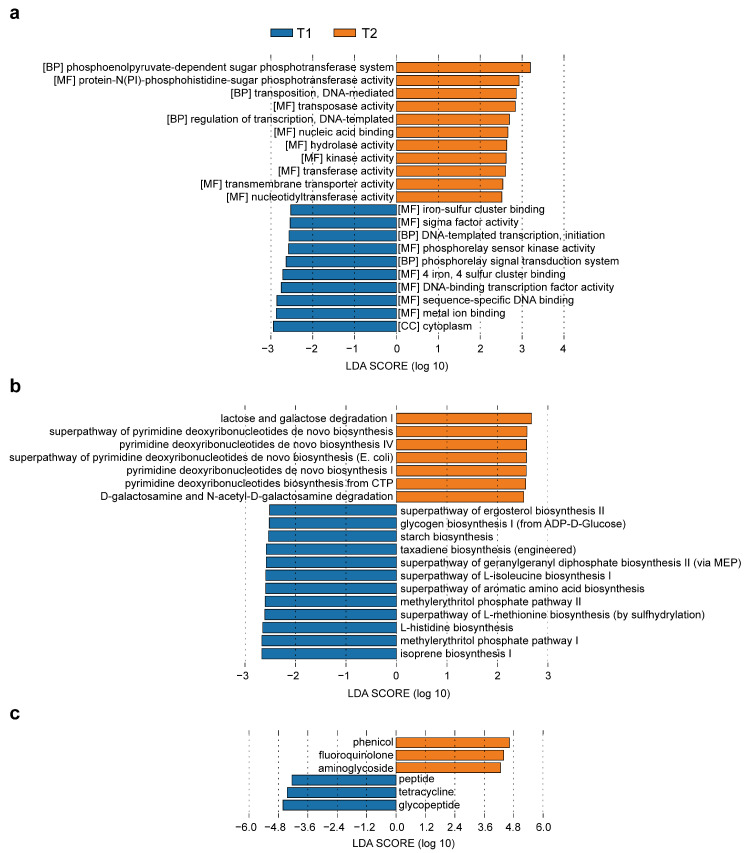
LEfSe Analysis of GO Terms, MetaCyc Pathways, and CARD Genes. (**a**) LEfSe analysis (LDA > 2.5) showing differentially expressed GO terms between T1 (preoperative) and T2 (postoperative) groups, covering biological processes (BP), cellular components (CC), and molecular functions (MF). (**b**) LEfSe analysis (LDA > 2.5) showing differentially expressed metabolic pathways between T1 and T2 groups. (**c**) LEfSe analysis (LDA > 2.5) identifying differentially expressed antibiotic resistance genes between T1 and T2 groups. Orange indicates genes enriched and upregulated post-surgery, while blue indicates genes enriched pre-surgery and downregulated post-surgery.

Based on the alterations observed in the multi-kingdom gut environment, we conducted PCA and NMDS analyses, and found significant changes in genes, metabolic pathways, and antibiotic resistance genes before and after surgery (Figure 6). Further LEfSe analysis identified differentially expressed functional genes, antibiotic resistance genes, and metabolic pathways between the pre- and post-surgical states (Figure A2).

The differentially expressed genes enriched post-surgery are involved in the following at the biological process (BP) level: the phosphoenolpyruvate-dependent sugar phosphotransferase system, DNA-mediated transposition, and DNA-templated regulation of transcription. At the molecular function (MF) level, they are associated with protein-N(PI)-phosphohistidine-sugar phosphotransferase activity, transposase activity, nucleic acid binding (Figure A2a). Post-surgery enriched metabolic pathways include lactose and galactose degradation I, the superpathway of pyrimidine deoxyribonucleotide de novo biosynthesis, and pyrimidine deoxyribonucleotide de novo biosynthesis IV. Pre-surgery enriched differential metabolic pathways include the methylerythritol phosphate pathway I, isoprene biosynthesis I, L-histidine biosynthesis, the superpathway of L-methionine biosynthesis (by sulfhydrylation) (Figure A2b). The three differentially enriched resistance genes post-surgery are phenicol, fluoroquinolone, and aminoglycoside, while those enriched pre-surgery are glycopeptide, tetracycline, and peptide (Figure A2c).

### 3.3. Correlation Analysis

#### 3.3.1. Correlations Between Multi-Kingdom Species

To determine whether there is a symbiotic relationship between multi-kingdom gut microbiomes, Spearman correlation analysis was performed to examine the correlations between different bacteria and fungi, archaea, and viruses before and after surgery.

**Figure 7:**
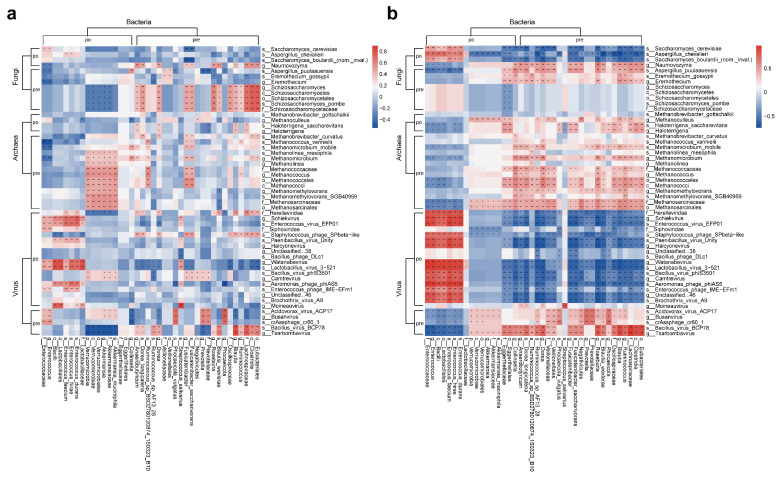
Correlations among multi-kingdom species. (**a**) Correlations between differentially enriched bacteria, fungi, archaea, and viruses in the pre-surgery group; this panel shows the correlations between pre-surgery and post-surgery enriched differential bacteria, fungi, archaea, and viruses in the pre-surgery group. (**b**) Correlations between differentially enriched bacteria, fungi, archaea, and viruses in the post-surgery group: this panel shows the correlations between pre-surgery and post-surgery enriched differential bacteria, fungi, archaea, and viruses in the post-surgery group. (Pre represents species enriched before surgery, Po represents species enriched after surgery, calculated using Spearman correlation). * *p* < 0.05; ** *p* < 0.01.

The results showed that the fungi enriched in the pre-surgery group such as *Schizosaccharomycetes* exhibited positive interactions with most pre-surgery enriched bacteria and negative interactions with post-surgery enriched *Akkermansiaceae*. This interaction was depleted after surgery. The pre-surgery enriched fungi *Eremothecium*, *Naumovozyma*, and *Aspergillus puulaauensis* showed positive interactions with most pre-surgery enriched bacteria, and these interactions became more pronounced after surgery (Figure 7a).

The post-surgery enriched fungi *Saccharomyces cerevisiae*, *Aspergillus chevalieri*, and *Saccharomyces boulardii (nom. inval.)* showed significant positive interactions with post-surgery enriched bacteria *Enterococcaceae* and *Bacilli*, while exhibiting negative interactions with most pre-surgery enriched bacteria (Figure 7b).

Most archaea had limited interactions with bacteria. However, positive interactions were observed between pre-surgery enriched archaea *Methanosarcinales*, *Methanomethylovorans*, *Methanococci*, *Methanolinea*, *Methanomicrobium*, and *Akkermansiaceae*. These positive interactions were depleted in the post-surgery group. In the post-surgery group, most archaea showed negative interactions with the post-surgery enriched bacteria *Enterococcaceae* and *Bacilli*, while showing positive interactions with pre-surgery enriched bacteria. These interactions were more abundant in the post-surgery group than in the pre-surgery group (Figure 7a,b).

In the pre-surgery group (Figure 7a), *Schiekvirus*, *Enterococcus virus EFP01*, *Watanabevirus*, and *Lactobacillus virus 3–521* were observed to have significant positive interactions with *Enterococcaceae*, *Bacilli*, and *Lactobacillales*, and these positive interactions were significantly enhanced post-surgery. In the post-surgery group (Figure 7b), most post-surgery enriched viruses showed significant positive interactions with post-surgery enriched bacterial species and significant negative interactions with pre-surgery enriched bacterial species. Meanwhile, pre-surgery enriched viruses showed positive interactions with pre-surgery enriched bacteria and negative interactions with post-surgery enriched bacteria.

#### 3.3.2. Multi-Kingdom Species and Genes, Metabolic Pathways, and Resistance Genes

**Figure 8:**
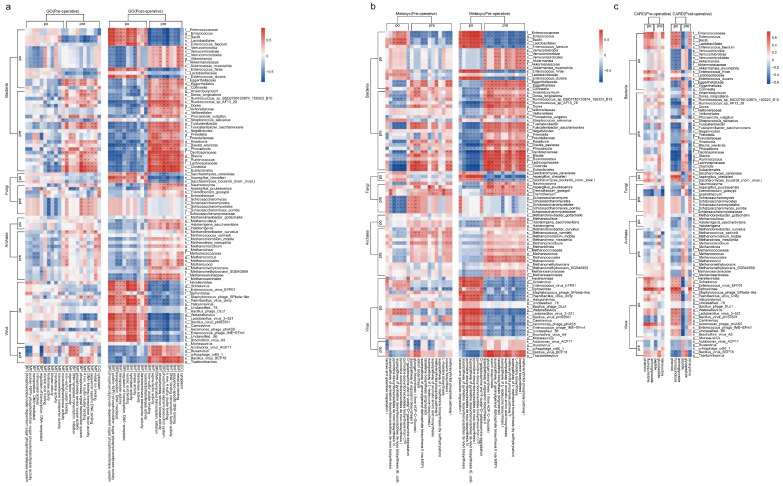
The correlation between multi-kingdom species and Metacyc and CARD. (**a**) Correlation between multi-kingdom species and GO genes (Pre represents species or genes enriched before surgery, Po represents species or genes enriched after surgery). (**b**) Correlation between multi-kingdom species and metabolic pathways (Metacyc) (Pre represents species or metabolic pathways enriched before surgery, Po represents species or metabolic pathways enriched after surgery). (**c**) Correlation between multi-kingdom species and resistance genes (CARD) (Pre represents species or resistance genes enriched before surgery, Po represents species or resistance genes enriched after surgery). * *p* < 0.05; ** *p* < 0.01.

We found that most genes, resistance genes, and metabolic pathways upregulated after surgery were significantly positively correlated with the post-surgery enriched bacteria *Enterococcus faecium*, *Enterococcus hirae*, *Enterococcus durans*, and *Bacilli*, however, there was a significant negative correlation with the post-surgery enriched bacteria *Akkermansia muciniphila* (Figure 8a–c). These upregulated genes also showed a negative correlation with most pre-surgery enriched bacteria. In contrast, pre-surgery enriched genes and metabolic pathways were almost entirely positively correlated with pre-surgery enriched bacteria, with these correlations becoming more pronounced post-surgery.

In the post-surgery group, genes and metabolic pathways that were upregulated post-surgery mostly showed a positive correlation with post-surgery enriched fungi, while genes that were downregulated post-surgery exhibited a negative correlation with most post-surgery enriched fungi. Most pre-surgery enriched fungi showed no significant correlation with genes and metabolic pathways enriched either pre- or post-surgery. However, the pre-surgery enriched fungi *Schizosaccharomyces pombe* had a significant positive correlation with pre-surgery enriched genes related to the phosphorelay signal transduction system, sequence-specific DNA binding, DNA-binding transcription factor activity, and 4 iron, 4 sulfur cluster binding in the pre-surgery group. These fungi also had a significant positive correlation with the metabolic pathways superpathway of ergosterol biosynthesis II, glycogen biosynthesis I (from ADP-D-Glucose), and starch biosynthesis in the pre-surgery group; however, no significant correlations were observed post-surgery.

Among archaea, post-surgery upregulated genes such as transposition, DNA-mediated, and transposase activity were negatively correlated with *Haloterrigena saccharevitans*, *Methanomicrobium mobile*. Meanwhile, post-surgery downregulated genes and metabolic pathways showed a positive correlation with these archaea.

In viruses, genes, resistance genes, and metabolic pathways that were upregulated post-surgery were mostly positively correlated with post-surgery enriched viruses and negatively correlated with viruses that decreased post-surgery. Conversely, genes downregulated post-surgery showed a negative correlation with viruses that increased post-surgery and a positive correlation with viruses that decreased post-surgery.

## 4. Discussion

In this study, we confirmed that the composition of multi-kingdom species in the gut significantly changed after coronary artery bypass grafting (CABG) surgery, accompanied by alterations in gene functions. The Spearman correlation analysis indicated widespread interactions between bacteria and fungi, bacteria and archaea, and bacteria and viruses, with significant correlations between changes in species and gene functions. This research will aid in uncovering the role of the gut microbiota in postoperative recovery and long-term prognosis following CABG, laying a solid foundation for future targeted therapies involving the gut microbiota after CABG surgery.

A cohort study found that, in patients undergoing cardiac surgery, the abundance of *Lachnospiracea*, *Blautia*, *Roseburia*, and *Dorea* significantly decreased during hospitalization, while *Enterococci* increased [6]. A pilot study revealed that in colorectal cancer patients undergoing surgery, at the phylum level, the relative abundance of *Proteobacteria* significantly increased, while *Bacteroidetes* decreased. At the genus level, the abundance of *Faecalibacterium*, *Roseburia*, *Ruminococcus*, and *Lachnospiracea_incertae_sedis* was reduced [7]. In our study, no significant changes were observed at the phylum level postoperatively, but similar changes were noted at the genus level, with reductions in *Roseburia*, *Ruminococcus*, and *Blautia Lachnospiracea*. Thus, certain microbial changes appear to be consistent after cardiac surgery.

In bacteria, we observed a postoperative decrease in beneficial bacteria and an increase in harmful bacteria. Specifically, the harmful bacteria such as *Enterococcaceae*, *Enterococcus*, *Enterococcus faecium*, *Enterococcus hirae*, and *Enterococcus durans*, showed elevated levels post-surgery, with most belonging to the *Enterococcus*. *Enterococcus* species are typically commensal in healthy states but can cause infections under certain conditions. *Enterococcus* is reported to be the second most common pathogen for both endogenous and exogenous hospital-acquired infections, particularly in immunocompromised patients, where it can cause infective endocarditis, urinary tract infections, abdominal infections, wound infections, and sepsis. Additionally, the impact of surgery on the immune system may increase patients’ susceptibility to *Enterococcus* infections, with *Enterococcus faecalis* and *Enterococcus faecium* being the most common species in clinical infections [8]. *Enterococcus faecalis* can cause chronic endocarditis, accounting for 10% of cases of valvular endocarditis. It can translocate through the gastrointestinal epithelium, leading to endogenous infections, invade vascular endothelium, and enter myocardial tissue, potentially inducing cell death and causing subendothelial microdamage in the heart [9,10].

In fungi, we found that the levels of *Schizosaccharomyces pombe*, which ranked highest in abundance preoperatively, significantly decreased post-surgery, while *Saccharomyces cerevisiae* and *Aspergillus chevalieri* significantly increased postoperatively. *Schizosaccharomyces pombe* was observed to have a significant positive interaction with anti-inflammatory bacteria such as *Fusicatenibacter saccharivorans*, *Eubacteriales*, *Blautia*, *Dorea*, and *Roseburia* preoperatively, as well as a significant positive correlation with the preoperatively enriched metabolic pathways glycogen biosynthesis I (from ADP-D-glucose) and starch biosynthesis. Thus, *Schizosaccharomyces pombe* may have beneficial functions in the human body. *Saccharomyces cerevisiae*, an ascomycete, is part of the human gastrointestinal, respiratory, and vaginal microbiomes. Early studies suggested that major cardiac surgery is a risk factor for susceptibility to *Saccharomyces cerevisiae*, with reports of three patients who died from fungal infections caused by this yeast after major cardiac surgery [11]. *Aspergillus chevalieri*, belonging to the genus *Aspergillus*, can cause various infections in immunocompromised individuals, including pulmonary, bloodstream, and brain infections. *Aspergillus chevalieri* has been detected in the pleural effusion of an infected patient [12] and can cause skin infections [13]. In our study, *Saccharomyces cerevisiae* and *Aspergillus chevalieri* were found to have antagonistic interactions with preoperatively enriched probiotic bacteria and positive interactions with postoperative bacteria associated with infections. Therefore, there is ample evidence to support the role of *Saccharomyces cerevisiae* and *Aspergillus chevalieri* in the development of infections following cardiac surgery.

Archaea are a unique group of microorganisms that occupy a small proportion of the gut microbiome and exhibit biological characteristics significantly different from those of bacteria and eukaryotes. Recent research has increasingly shown that archaea are present in the human gut, oral cavity, and skin, despite the fact that they are mainly found in harsh environments like high temperatures, high salinity, and high acidity. This suggests that archaea may be crucial to human health and the development of disease [14]. Sequences of *Methanobrevibacter spp.* have been detected in both the gastrointestinal and oral microbiomes, with a prevalence ranging from 38% to 95.5% [15]. Among them, *Methanobrevibacter smithii* and *Methanobrevibacter oralis* are considered to be the most prevalent archaea in the human gastrointestinal tract [14,16]. *Nitrosopumilus-SGB14899*, *Methanobrevibacter oralis*, *Methanobrevibacter millerae*, *Methanobrevibacter smithii*, *Methanomethylovorans-SGB40959*, *Methanolacinia petrolearia*, *Methanomassilicoccaceae archaeon DOK*, *Methanobrevibacter woesei*, *Methanogenium cariaci*, and *Methanoregula boonei* were among the top ten most abundant archaea in this study. Our investigation confirmed an extensive abundance of *Methanobrevibacter spp.* in the human gastrointestinal tract, which is in line with other research. Notably, *Methanomethylovorans-SGB40959*, which was enriched preoperatively, significantly decreased after surgery. *Methanomethylovorans* is a dominant methylotrophic methanogen [17] and showed positive interactions with the probiotic *Akkermansia muciniphila*, as well as with the probiotics *Ruminococcus* and *Dorea* postoperatively. Therefore, this archaeon may play a beneficial role during perioperative.

Bacteriophages, which are viruses that specifically infect and lyse certain bacterial species in the gut, form the majority of the human gut virome. The ratio of bacteriophages to bacteria in the gut microbiome can be as high as 1:1, reflecting their extensive presence [18]. The phages have the ability to control host immune responses, preserve the equilibrium of the gut microbiome, kill antibiotic-resistant bacteria specifically, and stop harmful bacteria from colonizing the body [19]. According to recent studies, the gut virome is linked to a number of illnesses, most notably autoimmune, metabolic, and inflammatory bowel disorders [20,21,22]. We found that the bacteriophage *Enterococcus-virus-EFP01*, which infects *Enterococcus* species, significantly increased after surgery. This phage had a 90% detection rate, making it the second most prevalent virus after surgery. It also showed a strong positive correlation with *Enterococcus*, which is consistent with previous studies [23]. The phage that infects *Staphylococcus aureus*, *Staphylococcus phage SPbeta-like*, was likewise concentrated and had a high abundance, second only to *Enterococcus-virus-EFP01*. After surgery, *Enterococcus* and *Staphylococcus aureus* are common pathogens associated with infections, and the increase in their corresponding bacteriophages observed in this study further supports the crucial role of bacteriophages in maintaining gut health.

Probiotic supplementation following surgery has been shown to improve gastrointestinal health. For instance, enteral probiotics significantly raised peripheral blood IgG levels and improved humoral and cellular immunity during the perioperative of colorectal cancer [24]. Furthermore, Yu et al. discovered that giving *Bifidobacterium* orally to patients with hepatocellular carcinoma following surgery increased recovery of liver function, reduced hospital stays, and increased one-year survival rates [25]. In another piece of RCT research involving heart valve surgery patients, those who took *Bifidobacterium animalis subsp. lactis LPL-RH* showed an increased relative abundance of beneficial gut genera such as *Bifidobacterium*, *Akkermansia*, *Faecalibacterium*, and *Gemmiger*, which led to a significant reduction in gastrointestinal symptoms and abdominal pain, without an increase in adverse events [26].

These findings suggest that modulating the gut microbiome through probiotics could offer clinical benefits, including improved immune function, reduced gastrointestinal discomfort, and enhanced recovery after surgery. We believe our results highlight the potential for microbiome-targeted interventions to improve patient outcomes in the perioperative period, especially in those undergoing major surgeries such as CABG.

## 5. Limitations

The most notable limitation of this study is its single-center design conducted only in China. Secondly, the sample size is not large. There are many factors that affect the intestinal flora. Due to the limited research samples and the single-center study, it is impossible to comprehensively analyze the impact of various factors on the intestinal flora. Further in-depth research is needed. Finally, changes in the intestinal flora are continuous. A study indicated that the intestinal microbial composition of patients undergoing cardiac surgery (including CABG, CABG + valve, and valve surgery) changed significantly during hospitalization. After discharge, it was found that the intestinal microbiota of most patients returned to the baseline composition, confirming the strong recovery ability of the intestinal microbiota [6]. This study currently only focused on changes in the perioperative intestinal microflora. Subsequent follow-up is still necessary to understand the recovery of the intestinal microbiome.

## 6. Conclusions

Dysbiosis of the gut microbiota has multifaceted impacts on health. Based on the metagenomic analysis of pre- and postoperative fecal samples from patients undergoing coronary artery bypass grafting (CABG), this study suggests that there is a disruption of bacteria, fungi, archaea, and viruses across multiple kingdoms in the gut microbiota, as well as functional dysregulation after surgery. In addition to improving our general knowledge of the postoperative gut microbiota, this study emphasizes the importance of probiotic therapy in treating gut microbiota dysbiosis in postoperative patients, which will help to lower postoperative complications, boost immunity, and facilitate recovery following CABG. Furthermore, this can be utilized with the planning of future research to precisely evaluate the possible gut microbiota biomarkers and develop targeted treatments for the prevention and management of postoperative gut microbiota dysbiosis.

## Figures and Tables

**Table 1:** Demographic characteristics of the study cohort at baseline.

	N = 40
Age (years)	62.55 ± 8.06
Male (N, %)	31 (77.5%)
BMI (kg/m^2^)	25.05 ± 3.42
Smoking (N, %)	21 (52.2%)
Drinking (N, %)	12 (30%)
Hypertension (N, %)	25 (62.5%)
Diabetes mellitus (N, %)	19 (47.5%)
Hyperlipidemia (N, %)	18 (45%)
Heart rate (bpm/min)	73 ± 14
Planned operation	
Coronary Artery Bypass Graft (CABG) (N %)	20 (50%)
Hybrid Coronary Revascularization (HCR) (N, %)	20 (50%)
Exposure antibiotics	
Days of β-lactamase (days)	7.35 ± 4.89
Chronic kidney failure (N, %)	2 (5%)
Postoperative sample collection time (days)	7.4 ± 4.0
Omeprazole/Pantoprazole (N, %)	40 (100%)
Anesthesia	
Intravenous Anesthetics (N, %)	12 (30%)
Intravenous + Inhalational (N, %)	28 (70%)
Systolic blood pressure (mmHg)	130 ± 15
Diastolic blood pressure (mmHg)	75 ± 11
Days of hospital(days)	20.5 (17.25, 25.75)
Syntax score	28.33 ± 6.50

Age, BMI, days of β-lactamase (days), postoperative sample collection time (days), systolic blood pressure, diastolic blood pressure are shown as mean ± SD; male, smoking, drinking, hypertension, diabetes mellitus, hyperlipidemia, coronary artery bypass graft (CABG), hybrid coronary revascularization (HCR), chronic kidney failure, omeprazole/pantoprazole, intravenous anesthetics, intravenous + inhalational are shown as percentage (%); length of stay (days) is shown as median (IQR). BMI, body mass index.

**Table 2:** Clinical characteristics of the study cohort.

Clinical Indicators	Preoperative	Postoperative	*p* Value
WBC (10^9^/L)	6.06 (5.69, 7.81)	10.47 (7.82, 11.70)	0.000
NE (10^9^/L)	3.82 (3.10,4.48)	9.02 (6.84, 10.52)	0.000
LY (10^9^/L)	1.84 ± 0.68	0.74 ± 0.36	0.000
SII	450.98 (325.22, 667.70)	2281.72 (1049.80, 3123.47)	0.000
Cr (umol/L)	68.80 (63.68, 80.20)	81.83 (66.18, 92.00)	0.001
UREA (mmol/L)	6.36 ± 2.07	5.42 ± 1.63	0.006
URIC (umol/L)	334.00 ± 71.27	245.92 ± 71.27	0.010
ALT (U/L)	23.50 (16.00, 31.00)	32.50 (23.25, 46.87)	0.017
AST (U/L)	21.00 (18.25, 34.00)	35.00 (26.25, 49.50)	0.003
TNI (ng/mL)	0.16 (0.00, 6.20)	16.98 (0.40, 778.10)	0.000

Continuous and normally distributed variables between the two groups were analyzed by Paired Sample *t*-test. Signed-rank test was applied to such data with non-normal distribution. WBC, white blood cell count; NE, neutrophilicgranulocyte; LY, Lymphocyte; SII, systemic immune-inflammation index, SII = Platelet Count (P) × Neutrophil-to-Lymphocyte Ratio (N/L); Cr, creatinine; ALT, alanine aminotransferase; AST, aspartate aminotransferase.

## Data Availability

The Metagenomic Sequencing data sets of all samples have been deposited in the Sequence Read Archive (SRA) database under the accession code PRJNA1165200 for multi-kingdom. The authors declare that all data supporting the study are accessible or can be obtained by contacting the corresponding author.
